# Representation of Linguistic Information Determines Its Susceptibility to Memory Interference

**DOI:** 10.3390/brainsci3031244

**Published:** 2013-08-07

**Authors:** Myra A. Fernandes, Jeffrey D. Wammes, Janet H. Hsiao

**Affiliations:** 1 Department of Psychology, University of Waterloo, 200 University Ave. West, Waterloo, ON N2L 3G1, Canada; E-Mail: jwammes@uwaterloo.ca; 2 Department of Psychology, University of Hong Kong, 604 Knowles Building, Pokfulam Road, Hong Kong, China; E-Mail: jhsiao@hku.hk

**Keywords:** memory, dual-task, language, representation, retrieval

## Abstract

We used the dual-task paradigm to infer how linguistic information is represented in the brain by indexing its susceptibility to retrieval interference. We measured recognition memory, in bilingual Chinese-English, and monolingual English speakers. Participants were visually presented with simplified Chinese characters under full attention, and later asked to recognize them while simultaneously engaging in distracting tasks that required either phonological or visuo-spatial processing of auditorily presented letters. Chinese speakers showed significantly greater memory interference from the visuo-spatial than phonological distracting task, a pattern that was not present in the English group. Such a pattern suggests that retrieval of simplified Chinese characters differentially requires visuo-spatial processing resources in Chinese speakers; these are compromised under dual-task conditions when such resources are otherwise engaged in a distracting task. In a secondary analysis, we showed the complementary pattern in a group of English speakers, whose memory for English words was disrupted to a greater degree from the phonological than visuo-spatial distracting task. Together, these results suggest the mode of representation of linguistic information can be indexed behaviorally by susceptibility to retrieval interference that occurs when representations overlap with resources required in a competing task.

## 1. Introduction

It is likely that we can use different codes, verbal, spatial, or a combination of the two, to represent different types of information. The dual-task technique—performing a distracting task simultaneously with a primary task—can be used to probe these representations. Based on the occurrence and magnitude of disruption in performance, relative to non-distracting conditions, one can infer whether concurrently performed tasks require an emphasis on the same processing resources or representational system as the primary task. Decrements in performance, termed interference effects, are observed when a common cognitive system, or brain area, is being overly taxed.

Previous research has shown that interference effects from dual-tasking (also known as divided attention (DA)) during memory retrieval are larger when there is overlap between the material used in the memory and concurrent distracting task [1,2,3,4,5,6,7,8]. Specifically, that body of work has found that during recall of a list of unrelated words, a visually presented word-based distracting task reduced memory by 30% compared to full attention conditions, whereas equally demanding digit-based distracting tasks led to only a 5%–13% reduction. 

Determining the key factors underlying this material-specific effect on memory can provide insight into the code used for representing and for retrieving items from memory. From these findings one could theorize that because words are primarily processed phonologically, distracting tasks that emphasize phonological processing will always produce more interference than distracting tasks that require other types of processing, be it numerical or visuo-spatial. The purpose of the current research is to determine the consistency of this generalization. It may hold true only for alphabetic languages (such as English) that rely on a high grapheme-phoneme correspondence [9]. In these languages, the smallest orthographic unit, a grapheme, corresponds to a phoneme in a predictable and patterned way, allowing a rapid transition from orthography to phonology. Language processing however is a learned way to represent information, which varies by culture. What would be the pattern of interference when the to-be-remembered stimuli are from a logographic language such as Chinese? This language differs from English in how words are represented, using a set of symbols or characters rather than letters. Characters do not encode phonemes, but rather full syllables and words; thus the correspondence between the printed stimulus and speech is not as clear [10,11,12]. 

Although some Chinese characters contain a phonetic component that typically provides partial information about character pronunciation, the mapping from the phonetic component to its pronunciation is at the syllable level and is not as fine-grained as the grapheme-phoneme correspondence in alphabetic languages [9,13]. Also, the relationship between the pronunciation of a character and its phonetic component is often ambiguous; only about 38% of the phonetic components convey consistent pronunciation information in the characters (a phonetic radical is consistent if all characters containing the phonetic component have the same pronunciation; see Feldman and Siok [14]; Zhou [15]; Hsiao and Shillcock [16]). Thus, the extraction of phonology from orthography in Chinese is typically not as transparent as that in alphabetic languages. In addition, due to the high incidence of homophones (up to 12 for some words [17]) in Chinese, words cannot be reliably distinguished by pronunciation, and the meaning of a word through semantic components is often attainable with little input from phonology [10,17,18]. The convergence of these factors leads those proficient in reading Chinese characters to rely more on a visuo-spatial mode of representation when processing these characters [19,20,21].

Studies of human visual processing suggest that particular attributes and categories of visual stimuli are represented and processed in specialized regions of extrastriate cortex [22]. The material-specific pattern of retrieval interference found in previous work [1,2,3,4,5,6,7,8] is in line with this. Competition is created primarily when the concurrent tasks both rely on word-processing regions in the brain. More recent work has refined this claim by suggesting representations of words or visuo-spatial information held in memory can be disrupted when the mode of processing (rather than material) overlaps with that required for retrieval of those items from memory. Specifically, Fernandes and Guild [23] tested whether episodic memory retrieval of words and spatial patterns was affected differently by concurrently performed distracting tasks which were distinguishable only by the type of processing required for each: phonological or visuo-spatial. They found an interaction such that a distracting task involving visuo-spatial processing of letters (*i.e.*, when capitalized, does it have any curved-lines?) had a more detrimental effect on recognition of spatial patterns than did a rhyme-based distracting task based on the same letters (*i.e.*, does the letter rhyme with long vowel “e”?) which had a more detrimental effect on memory for words. These results suggest it is similarity in processing requirements, and not materials per se, between the memory and distracting task, which leads to large memory interference.

The current study was designed to probe representations in memory by taking advantage of individual differences in how participants are able to encode and store information. We propose that these differences bias participants towards a particular mode of representation and that this subsequently determines their susceptibility to memory interference from different distracting tasks. Specifically, this study compared interference on a recognition task for simplified Chinese characters, when performed concurrently with either a phonological or visuo-spatial distracting task, in groups of participants who vary in their experience with such characters. 

Evidence from behavioral research supports the notion that Chinese word processing relies more on visual than phonological processing. For example, in a similarity judgment task, participants were shown a target word written as a simplified character, while it was simultaneously read aloud to them. They were then shown two characters, one of which was visually similar to the target, the other phonologically similar. Chinese participants favored the visual choice, suggesting they encoded the initially-presented Chinese character more visually than phonologically [9]. These findings are bolstered by correlational studies that indicate visuo-spatial abilities such as handwriting of characters, pseudo-character copying and picture drawing in Chinese children are more strongly correlated with reading ability of Chinese characters than are phonological discrimination tasks [19,21]. Such research suggests that Chinese characters are skewed towards a visuo-spatial mode of representation, while other research suggests representation of English words requires more phonological processing [24,25,26,27]. The current study explored whether memory retrieval interference patterns mirror this dissociation in the representation of Chinese characters *vs.* English words. 

Evidence from neuroimaging studies supports such a dissociation. While there exists considerable overlap between the activation patterns during reading of English and Chinese language, research has shown unique activation for Chinese character processing in the left lateral middle frontal cortex [12,27,28]. Not surprisingly, this region has been associated with visuo-spatial processing and visual working memory [29,30]. Importantly areas associated with memory for visuo-spatial information such as the right frontal pole (BA10/11), frontal operculum (BA 47/45), dorsolateral frontal gyrus (BA 9/44), and the superior and inferior parietal lobules (BAs 7, 40/39) ([12], p. 841) are all strongly implicated in Chinese, but not in English reading [12]. Dong *et al.* [31] attempted to isolate the fine spatial discrimination abilities required to process Chinese characters. Participants were asked to distinguish between orthographic legal characters and illegal pseudocharacters. They showed that spatial orientation of the character is automatically processed, and this visual processing is reflected in the activation of the previously mentioned network of frontal and parietal areas. 

We have presented some evidence to indicate that processing of the Chinese language has a relatively stronger visuo-spatial processing aspect than the English language, which relies more on phonology. The current study was designed to probe representations in memory by taking advantage of individual differences in how participants encode and store information. Such differences would influence susceptibility to memory interference from different distracting tasks. This comparison was accomplished by testing English monolinguals and Chinese-English bilinguals. Simplified Chinese characters were studied under full attention, and memory for these items was measured with a recognition task under conditions of either full attention (FA) or two different dual-task (divided attention (DA)) conditions, differing in their reliance on phonological or visuo-spatial processing requirements. Specifically, participants were exposed to one letter per recognition trial, to which they had to say yes or no aloud. For the phonological task, participants were to decide whether the letter rhymed with the long “e” sound, while the visuo-spatial task required them to decide whether the capitalized form of the letter had a curved line in it. These attention conditions were blocked such that participants studied a different set of characters for each attention condition. We hypothesized that the Chinese group would be much more susceptible to interference from the visuo-spatial than the phonological distracting task, as a result of their strong reliance on spatial processing for Chinese characters. We predicted that while the English-only group would be susceptible to interference as well, there would be no difference between the phonological and visuo-spatial distracting task conditions, due to a lesser reliance on visuo-spatial processing than the Chinese group. Additionally, research has shown that memory performance increases as a function of one’s familiarity with the type of stimulus [32], so performance in English-only participants should be overall lower due to inexperience with processing Chinese characters.

To further explore individual differences in word representations, we performed a secondary analysis, comparing our data set to that of Fernandes and Guild [23], in which memory for English words was assessed in monolingual English-speaking participants under conditions of full and divided attention. Importantly for the comparison, the paradigm (including distracting tasks) was identical to that of the current experiment, except for the to-be-remembered stimuli. Our comparison to this study allowed for a direct contrast of the influence of representational bias due to linguistic background on susceptibility to interference from different distracting tasks. We predicted that the English-only group from Fernandes and Guild [23] would show the complementary pattern to the Chinese-English group in the current study, such that their memory for English words would be more susceptible to phonological than visuo-spatial interference. This pattern should occur, due to the phonological bias in processing of English words. 

## 2. Results and Discussion

### 2.1. Primary Experiment: Effect of Processing Demands of Distracting Task on Memory Accuracy for Simplified Chinese Characters

#### 2.1.1. Recognition Memory Task Accuracy

The main effect of Order of conditions was non-significant, as were its interactions, thus analyses were conducted collapsing across this factor. A repeated measures ANOVA using accuracy (hit rate minus false alarm rate) as the dependent variable was conducted with Group (Chinese-English, English-only) as a between-participant and Condition (FA, DAP, DAV) as a within-participant factor. Mauchly’s test of Sphericity was violated, *X^2^*(2) = 6.05, *p* < 0.05. As such degrees of freedom were corrected using the Greenhouse-Geisser correction (ε = 0.888). There was a significant main effect of Group, *F*(1, 46) = 49.96, *p* < 0.001, characterized by the Chinese-English group (*M* = 0.53, *SE* = 0.03) scoring significantly higher than the English-only group (*M* = 0.81, *SE* = 0.03). There was also a significant main effect of Condition, *F*(1.78, 81.72) = 21.82, *p* < 0.001, which was explored using planned comparisons in the paragraph below. The interaction with group however, failed to reach significance, *F*(1.78, 81.72) = 0.26, *p* = 0.78.

As the apriori predicted difference of interest was between the DAP and DAV conditions in the two language groups, planned comparisons were performed to further delineate the nature of the main effects. These indicated that for each Group there was a significant main effect of Condition. For the Chinese-English group, the main effect of Condition (*F*(1.96, 37.57) = 13.96, *p* < 0.001) was driven by accuracy being higher in the FA than both the DAP, *F*(1, 23) = 10.91, *p* < 0.01, and DAV conditions, *F*(1, 23) = 26.03, *p* < 0.01. Notably, accuracy was significantly lower in the DAV than DAP condition in this group, *F*(1, 23) = 4.525, *p* < 0.05. For the English-only group, the main effect of Condition, *F*(1.63, 37.57) = 9.47, *p* < 0.01, was driven exclusively by accuracy being higher in the FA than in both the DAP, *F*(1, 23) = 15.86, *p* < 0.01, and DAV conditions, *F*(1, 23) = 18.25, *p* < 0.001, with no difference between the DAV and DAP conditions, *F*(1, 23) = 0.82, *p* > 0.05, *ns* (See Table 1). 

**Table 1:** Mean Accuracy in Chinese-English and English-only Groups Retrieving Chinese Characters, and English words in an English-only group (from Fernandes and Guild, 2009 [23]), in Each Memory Condition (Standard Deviations in brackets).

	Memory Condition		
	Full Attention	DA Phonological	DA Visuo-spatial
Chinese-English	0.93 (0.12)	0.80 (0.21)	0.70 (0.20)
English-only	0.68 (0.19)	0.49 (0.21)	0.42 (0.28)
English-only (from [23])	0.85 (0.15)	0.58 (0.18)	0.63 (0.22)

#### 2.1.2. Distracting Task Performance

The number of correct responses on the distracting task was divided by the total number of trials completed within each condition, in order to produce a performance score that was corrected for actual number of trials completed. Data were analyzed using a repeated measures ANOVA in a 2 × 2 × 2 ANOVA with Group (Chinese-English bilingual, English-only) as a between- and Attention (full, divided), as well as Distractor Task (phonological, visuo-spatial), as within-participant factors. The effect of Group was non-significant, *F*(1, 46) = 0.66, *p* > 0.05. Surprisingly, the main effect of Attention was also non-significant, *F*(1, 46) = 0.22, *p* > 0.05. There was a main effect of Distracting Task, *F*(1, 46) = 15.63, *p* < 0.001, which was qualified by a significant Group × Distracting Task interaction, *F*(1, 46) = 8.30, *p* < 0.01, with the English-only group performing significantly better on the phonological than visuo-spatial distracting task, *F*(1, 23) = 20.08, *p* < 0.001, though no such difference in accuracy emerged in the Chinese-English group, *F*(1, 23) = 0.69, *p* > 0.05 (see Table 2).

**Table 2:** Mean Distracting Task Performance in Chinese-English and English-only Groups, and in an English-only group (from Fernandes and Guild 2009 [23]) under Full (FA) and Divided Attention (DA) Conditions for Each Task (Standard Deviations in brackets).

Group	Phonological Task		Visuo-spatial Task	
	FA	DA	FA	DA
Chinese-English	0.94 (0.06)	0.98 (0.07)	0.96 (0.06)	0.92 (0.12)
English-only	1.00 (0.01)	0.98 (0.05)	0.89 (0.15)	0.88 (0.15)
English-only (from [23])	0.96 (0.05)	0.88 (0.11)	0.99 (0.02)	0.93 (0.07)

### 2.2. Secondary Analysis (Comparison with Fernandes and Guild [23]): Effect of Learned Mode of Representation on Memory Interference Patterns under Distraction

**Figure 1:**
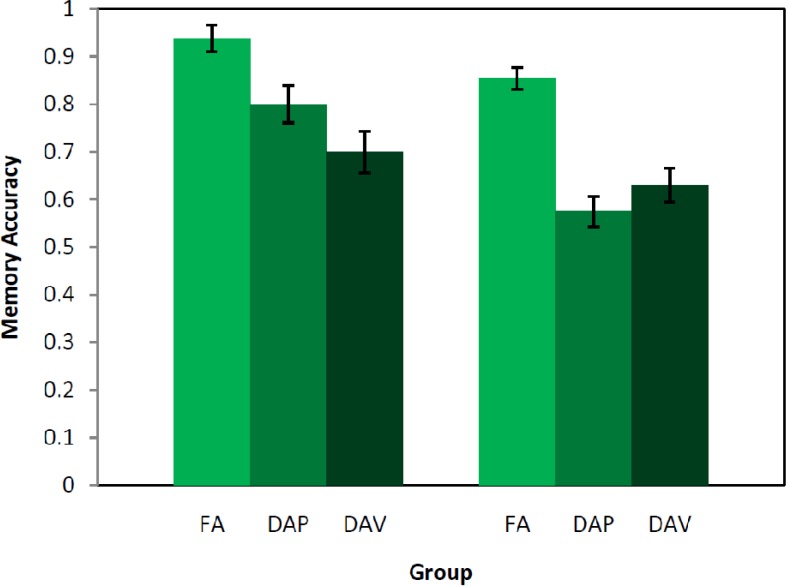
Memory accuracy for Chinese characters in Chinese-English Participants, and for English words in English-only participants from Fernandes and Guild (2009) [23], under FA = full attention, DAP = divided attention phonological, DAV = divided attention visuo-spatial retrieval conditions.

We found that the pattern of memory performance for Chinese characters, retrieved under distracting conditions, depended on language group status (See Figure 1). Chinese-English bilingual participants displayed significantly more memory interference from a visuo-spatial than phonological distracting task, whereas the English-only group showed similarly large memory interference in both distracting conditions. Such a pattern suggests that Chinese-English bilinguals relied differentially on visuo-spatial than phonological representations of the characters. This mode of representation was hampered under dual-task conditions when the distracting task also required the same processing resources. Such a suggestion follows from Klingberg’s [33] claim that interference is produced when there is substantial cortical overlap from competing tasks. 

While the pattern of results is suggestive, the statistical interaction between the language groups and the memory conditions did not reach significance. We aimed to show that individual differences in how participants are able to encode and store information influences the magnitude of memory interference experienced during its retrieval when performed under DA conditions with different distracting tasks. To offer further support for our claim, that learned mode of representation of information influences its susceptibility to interference, we compared the current set of data to those from our previous study (Fernandes and Guild [23]) in which English-only speakers had to retrieve English words (not Chinese characters) from memory under distracting conditions identical to those from our current study. Apart from using English words instead of Chinese characters, this experiment was exactly the same as our current study.

In this secondary analysis we tested the hypothesis that learned mode of representation of information influences its susceptibility to memory interference from like distracting tasks (*i.e.*, that both require the same processing resources). English speakers rely more on phonological processing to retrieve words written in English [12,34,35,36,37], and we have previously shown that their memory for words is more susceptible to interference from our phonological than visuo-spatial distracting task (Fernandes and Guild [23]). 

In comparing the pattern of memory interference in this English-only group when retrieving English words, to the Chinese-English group retrieving simplified Chinese, we could determine whether learned language-specific modes of representation would be reflected in our behavioural measure of memory interference under DA. English-only participants retrieving English words should show the complementary pattern of memory interference as the Chinese-English bilingual group retrieving Chinese characters. That is, the English-word-retrieval group should show greater interference in memory for English words from a phonological than visuo-spatial distracting task as English word representations rely preferentially on phonological [35] than visuo-spatial processes. This pattern would be reversed relative to Chinese-English bilinguals (in our primary experiment reported above) who show greater interference in memory for Chinese characters from a visuo-spatial than phonological distracting task. 

#### 2.2.1. Recognition Memory Task Accuracy

Data from the current experiment was compared with data from a nearly identical experiment on memory for English words under distraction (Fernandes & Guild, [23]). Memory performance calculated as hit rate minus false alarm rate was the dependent variable. Memory was for English words in the Fernandes and Guild [23] group and for Chinese characters in the Chinese-English group described earlier. A repeated measures ANOVA was conducted with Group (Chinese-English, English-only) as a between- and Condition (FA, DAP, DAV) as a within-participant factor. Mauchly’s test was not significant, so sphericity was assumed. There was a significant main effect of Group, *F*(1, 58) = 12.43, *p* < 0.01, such that the Chinese-English group (*M* = 0.81, *SE* = 0.03) performed significantly better than the English-only group (*M* = 0.69, *SE* = 0.02). There was also a main effect of Condition, *F*(2, 116) = 40.86, *p* < 0.001, which was explored using planned comparisons in the paragraph below Here the Group × Condition interaction was significant, *F*(2, 116) = 4.68, *p* < 0.05 (Figure 1).

In order to explore the main effect of Condition and the Group × Condition interaction, comparisons were performed. These indicated that for both groups there was a significant main effect of Condition (Chinese-English group: *F*(2, 46) = 13.96, *p* < 0.001; English group from Fernandes and Guild [1]: *F*(2, 70) = 36.18, *p* < 0.001). As already noted above, for the Chinese-English group accuracy in the FA condition was higher than in both DA conditions, and accuracy in the DAV was lower than in the DAP condition (see Results section above for statistics). The accuracy differences in the English group from Fernandes and Guild revealed a pattern in the opposite direction. The main effect of Condition was characterized by poorer performance in both DA conditions relative to FA (DAP: *F*(1, 35) = 67.57, *p* < 0.001; DAV: *F*(1, 35) = 50.13, *p* < 0.001) and a trend such that accuracy in the DAP was lower than in the DAV condition, *F*(1, 35) = 2.09, *p* = 0.16 (See Table 1 for means).

#### 2.2.2. Distracting Task Performance

Data were analyzed using a repeated measures ANOVA in a 2 × 2 × 2 ANOVA with Group (Chinese-English bilingual, English) as a between- and Attention (full, divided), as well as Distractor Task (phonological, visuo-spatial), as within-participant factors. The main effect of Group was non-significant, *F*(1, 58) = 0.59, *p* > 0.05. There was a significant main effect of Attention, *F*(1, 58) = 22.20, *p* < 0.001, such that performance was better overall under FA than DA conditions, but no main effect of Distracting Task, *F*(1, 58) = 0.86, *p* > 0.05. There were several 2-way interactions: Group × Distracting Task, *F*(1, 58) = 5.71, *p* < 0.05, Group × Attention, *F*(1, 58) = 17.54, *p* < 0.001, Distracting Task X Attention, *F*(1, 58) = 5.83, *p* < 0.05, and a 3-way Group × Distracting Task × Attention *F*(1, 58) = 11.32, *p* < 0.01 interaction.

For the Chinese-English group, there was no main effect of Distracting Task, *F*(1, 23) = 0.69, *p* > 0.05, or Attention, *F*(1, 23) = 0.10, *p* > 0.05, though the Distracting Task × Attention interaction was present, *F*(1, 23) = 15.07, *p* < 0.01. This was characterized by a larger reduction in performance, from FA levels, on the phonological than visuo-spatial distracting task. Conversely, for the English group, there was a main effect of Distracting Task, *F*(1, 35) = 8.57, *p* < 0.01 with a larger reduction in performance on the visuo-spatial than phonological task. There was also a main effect of Attention, *F*(1, 35) = 55.32, *p* < 0.001 with better performance under full attention than dual-task conditions, though again the Distracting Task × Attention interaction was not significant, *F*(1, 35) = 0.54, *p* > 0.05 (see Table 2 for means).

### 2.3. Discussion

The current study was designed to probe representations in memory by taking advantage of individual differences in how participants encode and store information. Such differences would influence susceptibility to memory interference from different distracting tasks. This comparison was accomplished initially by testing English monolinguals and Chinese-English bilinguals. Simplified Chinese characters were studied, and memory for these items was measured with a recognition task under either FA or two different DA conditions differing in their reliance on phonological or visuo-spatial processing requirements. The pattern of data suggests that Chinese-English participants relied differentially on visuo-spatial representations during retrieval of Chinese characters, whereas a comparison with English-only speakers retrieving English words suggested a differential reliance on phonological representations. Our findings revealed a significant interaction, suggesting that the degree to which distracting tasks interfere with memory performance differs depending on language group, the material to-be-remembered, and the overlap in processing demands in the distracting task. Specifically, the Chinese-English bilinguals displayed much more interference from the visuo-spatial than the phonological distracting task. The interference pattern in the English-only group was trending in the opposite direction. Our significant interaction is thus characterized by the two complementary interference patterns. That is, participants fluent in Chinese relied more on visuo-spatial than phonological processing resources when retrieving Chinese characters from memory, whereas English-only participants relied more on phonological than visuo-spatial processing to retrieve words written in English, as they were differentially hampered by the phonological than visuo-spatial distracting task. Such a finding is consistent with our hypothesis that individual differences in language experience may produce a bias towards one more of representation (e.g., visuo-spatial) over another (e.g., phonological).

While the Chinese and English languages both require aspects of phonological and visuospatial processing, there is evidence to suggest differences in the relative importance of each for the two languages. Specifically, English appears to require more phonological processing while Chinese more visuo-spatial. The interaction of group status by memory condition in our secondary analysis in this study is in line with this claim. 

Chinese logographs require a high degree of visuospatial scrutiny, as the orthographic appearance of characters does not directly dictate pronunciation of the word [10,11]. Behavioral research indicating a preference for selecting visually similar characters in a comparison task [9], as well as correlational research suggesting that spatial ability reliably predicts reading ability [19,20], point to the importance of visuo-spatial processing in Chinese character representation. Neuroscience research bolsters this claim, showing processing of Chinese characters is related to activation in brain regions typically associated with visuo-spatial processing and visual working memory [12,28,29,30]. Because of the visuo-spatial nature of Chinese character representations, it is not surprising that our study showed greater interference overall from a visuo-spatial than a phonological distracting task. Importantly, however, we showed that the Chinese-English group’s ability to retrieve Chinese characters was disrupted to a greater degree by the visuo-spatial distracting task, compared to an English-only group.

While visuo-spatial processing is important in Chinese character processing, phonology plays a necessary role in processing English words and accessing their meanings [25,38]. Though the role of phonology was previously thought to be inversely related with word frequency [39], there is convincing evidence that even for extremely common words, phonology is required to retrieve word meanings [25,40]. Accordingly, recognizing the studied English words in the Fernandes and Guild [23] study would require retrieval of a phonology-based representation of the word. As a result, it is not surprising that there was a high level of interference in this group from a phonological distracting task, which required overlapping processing resources.

When comparing retrieval of stimuli presented in each of these two languages, we see that language status of the participant, and hence ability to represent stimuli visuo-spatially in the case of Chinese and phonologically in the case of English words, determined the pattern of memory interference from distracting tasks that also required visuo-spatial or phonological processing. Because of the importance of visuo-spatial processing in Chinese, a visuo-spatial distracting task interfered more with the bilingual group’s retrieval of word representations, than did the phonological distracting task. Conversely, the English-only group’s (Fernandes and Guild, [23]) dependence on phonology resulted in the opposite trend, with phonological distraction being more costly.

According to a model put forward by Moscovitch and Umiltà [41], a memory trace consists of an ensemble of neurons in the neocortex that form the perceptual representation responsible for the content of the experience, needed during the ecphoric (memory reactivation) process. In the case of memory for English words, this likely includes orthographic, phonological, and semantic representations. That memory for English words would be interfered with to a greater degree by a phonological than visuo-spatial distracting task is in line with this claim, and suggests memory for words includes a significant phonological component. Our current study results suggest that the memory trace ensemble for Chinese characters would be weighted more heavily towards orthographic (visual) and less so on phonological representation. Taken together, these results suggest the mode of representation of linguistic information can be indexed behaviorally by susceptibility to retrieval interference that occurs when representations overlap with resources required in a competing task. 

## 3. Experimental Section

### 3.1. Primary Experiment: Effect of Processing Demands of Distracting Task on Memory Accuracy for Simplified Chinese Characters

#### 3.1.1. Participants

Forty-eight undergraduate students were selected from two separate institutions based on their language background. They were provided with course credit or monetary remuneration for their participation. Twenty-four monolingual English participants ranging in age from 18 to 25 years (*M* = 20.04, *SD* = 1.83) were undergraduates from the University of Waterloo in Canada and had no prior Chinese language experience. Their education ranged from 13 to 20 years (*M* = 14.46, *SD* = 1.72). Another group of 24 participants were bilingual Mandarin-English undergraduates from Mainland China attending the University of Hong Kong, ranging in age from 18 to 24 years (*M* = 20.33, *SD* = 1.63), with education ranging from 13 to 19 years (*M* = 14.81, *SD* = 1.77). Participants in this group were required to complete a basic reading test to indicate their ability to parse both meaning (*M* = 99.2%, *SD* = 1.6%) and pronunciation (*M* = 99.6%, *SD* = 1.0%) from Chinese characters; performance validates their language grouping. The groups did not differ in age, *t*(46) = −0.58, *p* > 0.05 or education *t*(46) = −0.70, *p* > 0.05.

#### 3.1.2. Materials

**Figure 2:**
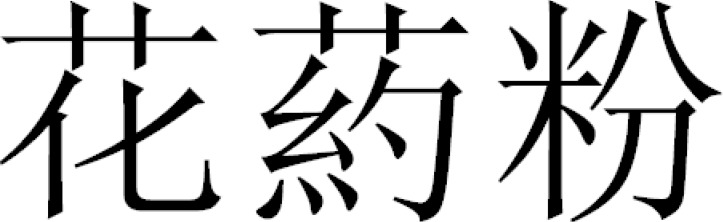
Three examples of simplified Chinese characters used as stimuli.

*Chinese Character Memory Task.* The stimuli for the memory task were composed of 70 single characters, each written in their corresponding simplified Chinese character. Characters had a frequency between 2 and 5921 per 662,700 occurrences and a mean number of strokes 8.24 [42]. These were divided into a single practice list of 10 characters, as well as 3 experimental lists of 20 characters each. Within each list, half of the characters were randomly chosen to be targets, while the other half were used as lures on the recognition test. Characters appeared approximately 6 cm high and 8 cm wide on a computer screen (See Figure 2 for samples of characters).

*Distracting Task.* The same stimuli were used for both the phonological and visuo-spatial distracting tasks, and consisted of 23 of the 26 letters of the English alphabet (omitting A, M, and W). These were recorded by a female speaker as separate audio files (.wav) via a microphone using Sound-Designer II software (Palo Alto, CA, USA). Audio files were created such that each .wav file was approximately 1500 ms in duration.

#### 3.1.3. Procedure

Participants were seated in front of a computer monitor, and the experiment was administered using E-prime v1.1 software (Psychology Software Tools Inc., Pittsburgh, PA, USA) via an IBM computer. Instructions were presented in English on the screen, as well as read aloud by the experimenter. The session began with a practice phase to familiarize participants with the tasks.

*Practice Phase*. Participants were asked to study 5 Chinese characters for later recognition. Stimuli were presented one at a time in the center of the screen for a duration of 3500 ms, followed by a fixation cross for 500 ms. After a brief delay participants were subsequently instructed to complete the recognition task, which consisted of pressing the “m” key to identify stimuli they remembered seeing in the study phase. The 5 characters from study were presented intermixed randomly amongst 5 lure characters. Each stimulus was presented in the center of the screen for 1500 ms, during which time the participants were required to make their response. Between each stimulus a fixation cross was presented for 500 ms.

Participants also completed a practice session for the phonological and visuo-spatial distracting tasks. For both tasks, participants heard a female voice speaking a list of 15 letters aloud. Each audio file was played for 1500 ms in duration followed by 500 ms of silence. Participants were asked to make a “yes” or “no” response immediately after presentation of each letter, and the experimenter recorded their responses. For the phonological task participants had to respond “yes” if the letter presented rhymed with the long “e” vowel. For example, letters requiring a “yes” response are B, C, D, E, G, P, T, and V. If the letter did not rhyme with the long “e” vowel, the participant responded “no.” Thus, the task required the participant to make phonological decisions about the letters. Five letters requiring a “yes” response were randomly selected, and presented randomly amongst 10 randomly selected letters requiring a “no” response. In the practice phase of the visuo-spatial decision task, participants had to imagine the letter in their “mind’s eye,” and respond “yes” if the letter contained a curved line, when in its capitalized form. Participants were instructed to think of the capitalized alphabet. For example, correct “yes” responses would be to the letters B, C, D, G, P, J, O, P, Q, R, S, and U). If the letter, when capitalized, did not contain a curved line, the participant responded “no.” To clarify the task, the experimenter referred to the letters on the computer keyboard as illustrating how participants should visualize the alphabet they based their decisions on. As in the phonological distracting task, the list consisted of five letters requiring a “yes” response that were randomly selected, and presented randomly amongst 10 randomly selected letters requiring a “no” response. Participants were encouraged to ask for clarification on any of the tasks if needed.

*Experimental Phase*. Participants first completed a baseline measure of one of the distracting tasks, followed by three memory conditions, and lastly a baseline of the other distracting task. The order of the baseline distracting task was counterbalanced across participants, as was the order in which they completed the memory conditions. The baseline tasks consisted of lengthened versions of the phonological distracting task or the visuo-spatial distracting task described above. Each of these consisted of 20 trials in which 8 required “yes” responses, and the remaining 12 required “no” responses. All other aspects of the task were identical to the practice phase.

For each of the memory conditions, encoding during the study phase was performed under full attention. In the FA condition, participants completed the recognition task without any distracting task. In the divided attention phonological (DAP) and divided attention visuo-spatial (DAV) conditions participants had to make their recognition memory decisions to the simplified Chinese characters, whilst simultaneously making decisions to letters in a distracting task. For each memory condition, participants began by studying 10 different Chinese characters. As in the practice phase, they were asked to memorize these for a later memory test. Immediately following study, a recognition test was given in which participants were presented with 20 characters: 10 from study presented randomly amongst 10 new “lure” characters, and participants pressed the “m” key to identify characters that were “old” (from the study phase). For all of the experimental phase recognition tests, characters presented for 1500 ms followed by a fixation cross for 500 ms, as in the practice recognition test. A short break of 1 to 2 min was given after each recognition condition. 

In the FA condition the recognition test was performed with no distracting tasks. In the DA conditions (DAP and DAV) for each group, participants had to make their recognition memory decisions, whilst simultaneously making decisions to letters in the distracting task. The onset of the first item in the visually presented recognition task and the auditorily presented distracting task was simultaneous. Participants were told to place equal effort on performing the memory and distracting task. During recognition in the DAV condition, participants simultaneously made key press responses to old items whilst making “yes/no” responses aloud if the spoken letter contained a curved line. During recognition in the DAP condition participants simultaneously made keypress responses to old items whilst making “yes/no” responses aloud if the spoken letter rhymed with the long vowel “e”. The importance of allocating equal attention to each of the tasks was emphasized. 

### 3.2. Secondary Analysis (Comparison with Fernandes and Guild [23]): Effect of Learned Mode of Representation on Memory Interference Patterns under Distraction

#### 3.2.1. Participants

Participants in Fernandes and Guild [23] were 36 English speaking undergraduate students (19 male) from the University of Waterloo, whose ages ranged from 18 to 24 years (*M* = 20.22, *SD* = 1.49). These data were compared with the 24 Chinese-English bilinguals from the primary experiment. 

#### 3.2.2. Materials

*Chinese character memory task*. As described above.

*English word memory task*. The word stimuli for the memory task in Fernandes and Guild [23]) were composed of 112 nouns, each written in English letters. Words had a frequency between 40 and 100 per million and a mean word length of 5.84, based on the Frequency Analysis of English Usage [43]. These were divided into a single practice list of 16 words, as well as 3 experimental lists of 32 words each. Within each list, half of the words were randomly chosen to be targets, while the other half were used as lures. 

*Distracting tasks*. As described above.

#### 3.2.3. Procedure

The only differences between the Fernandes and Guild (2009) [23] study and the current one are that memory was for English words rather than Chinese characters, and that the number of words in the study phase was slightly larger in the former study (32 English words per list rather than 20 Chinese characters).

## 4. Conclusions

The current study was designed to probe representations in memory by taking advantage of individual differences in how participants encode and store information. Such differences influence susceptibility to memory interference from different distracting tasks. The results of our experiment showed that individual differences in how stimuli are represented influenced patterns of memory interference from distracting tasks. In our primary experiment, the bilingual group displayed a higher cost from a visuo-spatial distracting task relative to a phonological task, when retrieving Chinese characters from memory; a pattern which was not mirrored by the English-only group. Such a result suggests their memory trace is based more in visuo-spatial than phonological representations. 

Through secondary analysis with a previous study [23], our experiment compared susceptibility to interference for words presented in English *vs.* Chinese. We showed a significant interaction, which was characterized by the two groups displaying complementary interference patterns in response to distracting tasks. The memory trace for English words, in English speakers was trending such that performance was more susceptible to interference from the phonological than visuo-spatial distracting task, while interference for Chinese characters in Chinese participants significantly displayed the opposite pattern. This finding provides some early evidence suggesting that language experience mediates the manner in which memory trace is encoded.

It is clear that language is an important contributor to the organization of one’s memory. Several studies have shown that Chinese and English processing activate diverging neural networks [24,25,26,27,28,29,30,31], and our findings suggest that this directly affects the way a memory trace is encoded. People are not typically in situations where they can focus their attention entirely on one task, as there are constant multi-sensory distractions in everyday life. In these situations, where attentional resources are overly taxed, it is important to know the limitations of our memory system, and the dual-task paradigm provides a way to test these limitations. It appears that language experience influences the types of multi-tasking conditions in which one is able to effectively cope, and our study identifies the conditions in which memory breaks down. In the future, such knowledge can not only inform effective learning and testing strategies for students, but also ways in which aging populations can efficiently use their processing resources to benefit memory.

These findings are in line with Moscovitch and Umiltà’s [41] model, which proposes that a memory trace is composed of a set of cortical neurons that create a perceptual representation of the information or experience. Representations can be created by individuals based on their language status, and may have a relatively heavier reliance on either phonological or visuo-spatial processing resources. The nature of the representations can directly affect one’s ability to retrieve this information under dual-tasking conditions.
